# Pancreatoscopy assists in the diagnosis of malignant transformation in chronic pancreatitis

**DOI:** 10.1055/a-2462-1702

**Published:** 2024-11-18

**Authors:** Tingting Yu, Zengfang Hao, Yankun Hou, Lichao Zhang, Jiao Tian, Senlin Hou

**Affiliations:** 1 71213 Biliopancreatic Endoscopic Surgery, The Second Hospital of Hebei Medical University, Shijiazhuang, China; 2 71213 Pathology, The Second Hospital of Hebei Medical University, Shijiazhuang, China

Patients with chronic pancreatitis have an increased risk of pancreatic cancer 1 . Unfortunately, there are no effective screening strategies for early detection of pancreatic cancer in these patients 2 . An active role for pancreatoscopy has been shown in the diagnosis of intraductal papillary mucinous neoplasm (IPMN) 3 , in lithotripsy for pancreatic duct stones 4 , and in laser stricturoplasty 5 . We describe the case of a patient with chronic pancreatitis in whom malignant transformation was detected by pancreatoscopy, so possibly we should emphasize the diagnostic role of pancreatoscopy in patients with chronic pancreatitis.

A 33-year-old man was admitted to our hospital with intermittent abdominal pain. He had a 10-year history of drinking alcohol and had been diagnosed with acute pancreatitis at another hospital 7 years previously. Magnetic resonance imaging (MRI) showed chronic pancreatitis with stones in the main pancreatic duct ( Fig. 1 ). We chose endoscopic ultrasonography (EUS) for further examination ( **Video 1** ). EUS revealed dilatation of the pancreatic duct, with stones in the pancreatic duct at the head of the pancreas ( Fig. 2 **a** ) and hypoechoic nodules in the pancreatic duct wall near the stones ( Fig. 2 **b** ), and no “fish mouth” appearance at the major papilla ( Fig. 2 **c** ). To further clarify the diagnosis, endoscopic retrograde cholangiopancreatography (ERCP) was performed and a novel peroral pancreatoscope (eyeMax Pancreatoscope System Digital Controller; Micro-Tech, Nanjing, China) was used subsequently to explore the pancreatic duct ( Video 1 ).

Pancreatoscopy-aided diagnosis of malignant transformation in a patient with chronic pancreatitis.Video 1

**Fig. 1:**
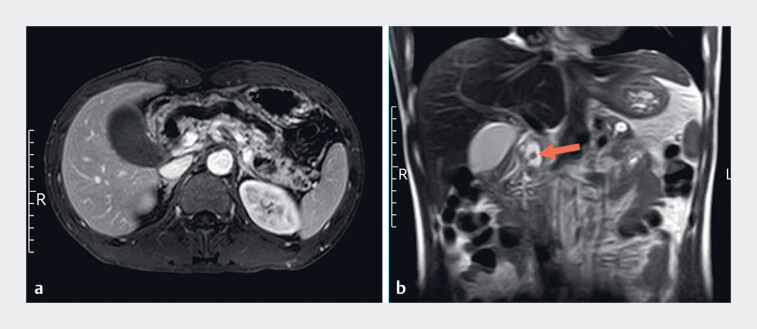
Magnetic resonance imaging (MRI) showed chronic pancreatitis with main pancreatic duct stones in a 33-year-old man with intermittent abdominal pain. **a** Dilatation of the main pancreatic duct. **b** Pancreatic duct stone (arrow) at the head of the pancreas.

**Fig. 2:**
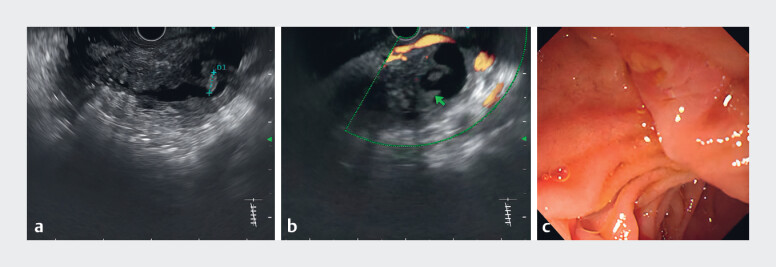
**a** Endoscopic ultrasonography (EUS) revealed pancreatic duct stones at the head of the pancreas, including a stone 8.8 mm in diameter. **b** Hypoechoic nodules (arrow) near the stones. **c** Endoscopically there was no “fish mouth” appearance or mucus at the major papilla.

Several stones were clearly visible in the main pancreatic duct (MPD) and in partially wide side branches ( Fig. 3 **a, c** ). Proliferative lesions with a fragile surface were seen in the MPD at the head of the pancreas ( Fig. 3 **b** ). No significant mucus was observed in the pancreatic duct. We then performed pancreatoscopy-guided biopsy using a biopsy forceps and successfully removed MPD stones. Pathological examination revealed adenocarcinomatous tissue originating from the epithelium of the pancreatic duct ( Fig. 3 **d** ). A final diagnosis was made of chronic pancreatitis with regional development of cancer .

**Fig. 3:**
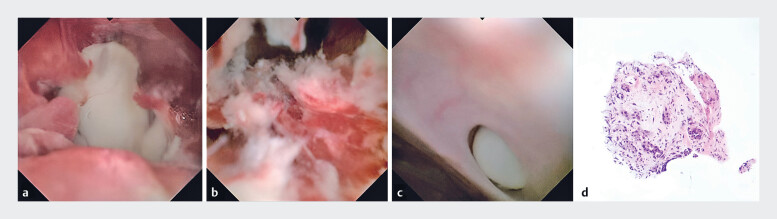
**a** Stones in the main pancreatic duct and the hyperplastic tissue surrounding them. **b** The surface of the hyperplastic lesion is fragile and rich in tortuous blood vessels. **c** A stone in the wide branch duct. **d** Pancreatoscopy-guided biopsy obtained adenocarcinomatous tissue that originated from the epithelium of the pancreatic duct.

Endoscopy_UCTN_Code_TTT_1AR_2AK

## Notes

Endoscopy E-Videos https://eref.thieme.de/e-videos
*E-Videos* is an open access online section of the journal *Endoscopy* , reporting on interesting cases and new techniques in gastroenterological endoscopy. All papers include a high-quality video and are published with a Creative Commons CC-BY license. Endoscopy E-Videos qualify for HINARI discounts and waivers and eligibility is automatically checked during the submission process. We grant 100% waivers to articles whose corresponding authors are based in Group A countries and 50% waivers to those who are based in Group B countries as classified by Research4Life (see: https://www.research4life.org/access/eligibility/ ). This section has its own submission website at https://mc.manuscriptcentral.com/e-videos .
